# Associations Between Eating Disorders and Sociodemographic Factors in Adolescent Patients Since the Start of the COVID-19 Pandemic

**DOI:** 10.3390/children12060730

**Published:** 2025-05-31

**Authors:** Janet Lee, David Miller, Paulina Rugart

**Affiliations:** Lewis Katz School of Medicine, Temple University, Philadelphia, PA 19122, USA

**Keywords:** eating disorders, adolescents, health disparities, COVID-19

## Abstract

Background/Objectives: The COVID-19 pandemic has been associated with significant increases in mental-health-related concerns in adolescents, including eating disorders. Disparities in screening, diagnosis, and treatment impact adolescents with eating disorders. This study aimed to describe the patterns in the prevalence and the associations between eating disorder diagnoses and demographic factors in adolescent patients since the start of the COVID-19 pandemic. Methods: We performed a retrospective cohort study examining adolescent patients (aged 12 to 21) with an eating disorder (ED) diagnosis documented between January 2019 and July 2023 using Epic Systems Corporation’s Cosmos, a de-identified dataset aggregated from electronic health record (EHR) data. We examined the differences in demographic factors by utilizing chi-square and Kruskal–Wallis rank sum tests. Results: A total of 82,435 distinct adolescent and young adult patients with eating disorder diagnoses were included in the analytical dataset. The overall prevalence of EDs has increased since 2019. The median age of patients with an ED decreased between 2019 and 2023. There was a decrease in other eating disorder diagnoses and an increase in avoidant-restrictive food intake disorder (ARFID) during the study period. There was a decrease in the proportion of individuals who identified as White and an increase in the proportion of adolescents from historically minoritized racial and ethnic groups (i.e., African American or Black and Hispanic). There was also an increase, during this study period, in the proportion of adolescents with an ED diagnosis who were from more socially vulnerable communities. Conclusions: Our study describes the changes in the prevalence of sociodemographic factors in adolescent patients with EDs since the start of the COVID-19 pandemic. Further studies should address screening, diagnostic, and treatment barriers for EDs in historically underserved communities.

## 1. Introduction

Eating disorders (EDs) are significant, potentially life-threatening illnesses that significantly affect the physical and psychological development of adolescents and young adults [1]. Eating disorders affect every organ system and can lead to serious complications related to malnutrition, weight changes, and purging behaviors [1]. Eating disorders can also seriously impact social functioning as well as lead to medical complications, mortality, and suicide [1,2].

The prevalence EDs has been rising in adolescents and young adults globally, especially since the start of the COVID-19 pandemic [3,4]. This rise has been attributed to multiple factors. The crisis in children’s mental health, an increase in maladaptive methods of asserting control, social isolation, and increases in stress and anxiety are considered to be the major drivers of the rise in the prevalence of eating disorders [3,4,5,6].

Studies have demonstrated that eating disorders affect patients of all sexes, races, and ethnic groups [7,8,9]. Additionally, research has shown that EDs are present across differing levels of socioeconomic status [10,11]. Despite this information, biases are often perpetuated, describing an eating disorder as a “disease of affluence”, or predominantly impacting those who identify as White [2,9,12]. Eating disorders are also mischaracterized as only affecting non-males with average or normal body sizes [1]. These biases have significant implications for diagnosis and treatment.

Screening is essential for early intervention, as this can decrease the adverse impacts of eating disorders on health and improve adult ED recovery rates [11,13]. Eating disorders have been found to be under-identified in young people from lower-income families [11]. Additionally, screening practices in public mental health systems and primary care settings are suboptimal [11]. Adolescents and young adults from lower-income families are also undertreated for EDs. Although ED treatment is covered by public health insurance (i.e., Medicaid) in the United States, the terms of payer coverage and administrative hurdles favor intensive medical interventions, while denying coverage for behavioral health treatments [11]. Additionally, the access to family-based therapy, an effective, evidence-based treatment for EDs, is extremely limited for patients with public insurance [11,13].

To the best of our knowledge, studies have yet to address the sociodemographic patterns of adolescents and young adults with eating disorder diagnoses since the start of the COVID-19 pandemic. Our study is unique in that it performed a novel socioeconomic trend analysis of adolescent patients with eating disorders during this period. We expected that the overall prevalence of eating disorders had increased. Additionally, given the disproportionate impacts of the pandemic on individuals from historically marginalized groups [14], we expected that adolescents and young adults from minoritized racial and ethnic groups and lower socioeconomic status had an increased eating disorder prevalence. This study aimed to describe the patterns in the prevalence and the associations between eating disorders and sociodemographic factors in adolescents and young adults since the start of the COVID-19 pandemic. We hypothesized that the prevalence of eating disorders increased in the years following the start of the COVID-19 pandemic and that a higher proportion of individuals from underserved communities had eating disorder diagnoses during this time.

## 2. Materials and Methods

### 2.1. Study Sample and Setting

We performed a retrospective cohort study examining adolescent patients (ages 12 to 21) with a new eating disorder diagnosis documented between January 2019 and July 2023 using Epic Systems Corporation’s Cosmos [15]. Cosmos is a HIPAA-defined limited dataset. Cosmos was created in collaboration with a community of Epic health systems and represents more than 299 million patient records from 1714 hospitals, more than 40,100 clinics from all 50 American states, Lebanon, and Saudi Arabia [15]. Health systems participating in the Cosmos network contribute data at differing times to the dataset. Th source data within Cosmos were originally collected within contributing health systems’ electronic health records (EHRs). EHR data may have several limitations, including being inaccurate or incomplete, and the availability of particular measures may be lacking, especially those related to behavioral health [16,17].

Prior to analysis, we completed an in-depth cleaning process to ensure the integrity of the dataset. Missingness was assessed for each variable. Any cases with missing values in our study measures were excluded using listwise deletion. Given the limitations of the dataset, this approach was chosen to minimize the risk of bias, as missing data were assumed to be missing complete at random. A total of 6.4% of the data were missing across our study variables. No imputation methods were utilized, as the amount of missingness was deemed to be sufficiently low to avoid introducing bias to our results. We followed standard protocols for outlier management and excluded any outliers beyond three standard deviations from the mean. All variables were consistently coded and checked for errors prior to proceeding with data analysis.

Inclusion criteria included any adolescent patient (ages 12 to 21) with a new eating disorder (ED) diagnosis documented between January 2019 and July 2023. Subjects were only sampled once. Diagnoses for ED were identified by aggregating the following ICD-10-CM codes: F50.0 (anorexia nervosa); F50.2 (bulimia nervosa); F50.82 (avoidant-restrictive food intake disorder (ARFID)); F50.81 (binge eating disorder); F50.9 (eating disorder not specified). Exclusion criteria included patients who were missing study-measured data. ICD-10s are limited in their ability to capture differences in subtype and severity, lack specific criteria for atypical conditions, and overlap between multiple eating disorder diagnoses [18]. Despite these limitations, we utilized ICD-10 codes given the availability of this information with Cosmos.

This study was not deemed to constitute as human-subject research and did not require IRB approval or review.

### 2.2. Study Measures

Study variables included patient age, sex, race, ethnicity, year of initial diagnosis noted in Cosmos, and social vulnerability index (SVI). Sociodemographic variables were selected a priori based on availability within Cosmos. Though the financial class variable, a measure noting patients’ insurance types, was available in Cosmos; however, due to the large amounts of missing data, this measure was not included in our dataset.

The social vulnerability index (SVI), a variable used to describe the socioeconomic factors that contribute to communities being more adversely affected by public health stressors that contribute to disease and harm [19], was utilized as a proxy for patients’ social circumstances. At the time of data extraction from Cosmos, the SVI value was the only available variable that could be utilized as a proxy for socioeconomic status. The SVI measure within the Cosmos dataset is a continuous variable. SVI values are noted to be between 0 and 1. Lower SVI values indicate lower degrees of social vulnerability [19]. The SVI was first converted from a numerical value into a categorical variable and then grouped by quartile to improve the interpretability of the results. In this study, we defined individuals in SVI quartiles 3 and 4 as higher social vulnerability and those in SVI quartiles 1 and 2 as lower social vulnerability.

For race, we aggregated the Asian, Native Hawaiian, and Pacific Islander groups racial groups into one group entitled, “AAPINH”, in alignment with Epic’s Cosmos privacy and security policies [15]. Policy guidance supported the disaggregation of race data for individuals from the Asian American and Native Hawaiian/Pacific Islander communities [20,21], but, because of the low patient numbers in the disaggregated data, subgroups were combined in this study.

### 2.3. Statistical Analyses

Descriptive statistics were utilized to determine the median and interquartile range for continuous variables for adolescent patients diagnosed with an eating disorder since 1 January 2019, while frequencies and percentages were used for categorical variables. Kruskal–Wallis rank sum tests were employed to compare differences in our dataset between groups for continuous variables (i.e., age) for non-normally distributed data. Chi-square tests were used to evaluate differences between categorical variables (i.e., race, SVI, sex, and ethnicity). *p*-values of 0.05 were considered statistically significant. Data were analyzed in the R environment (R version 4.2.3, http://www.r-project.org, accessed on 5 May 2025) [22].

## 3. Results

### 3.1. Descriptive Statistics

A total of 88,029 unique adolescent patients with an eating disorder diagnosis were identified within the Cosmos dataset. Cases that were missing data from the study variables (i.e., age, sex, race, ethnicity, diagnosis, SVI) were excluded from the dataset. A total of 82,435 cases were included in the analytical dataset. Table 1 presents the descriptive statistics of the total sample.

The median patient age was 17 years (IQR 15–19). A total of 83% (*n* = 68,486) of the population identified as female, 17% identified as male (*n* = 13,888), while <0.1% identified as other/unknown sex; 78% (*n* = 64,618) of the population identified as White, 10% (*n* = 8622) identified as African American or Black, 5.9% (*n* = 4860) identified as other, 4.1% (*n* = 3352) identified as AAPINH, while 1.2% (*n* = 983) of the population identified as American Indian or Alaska Native. A total of 79% (*n* = 65,448) of individuals in the analytical dataset identified as not Hispanic or Latino. Of the patients, 58% (*n* = 47,559) had a diagnosis of eating disorder not specified, 20% (*n* = 16,242) had a diagnosis of anorexia nervosa, 9.7% (*n* = 8006) of patients had a diagnosis of ARFID, 6.8% (*n* = 5627) had a diagnosis of bulimia nervosa, and 6.1% (*n* = 4991) of patients had a diagnosis of binge eating disorder. Of the patients, 40% (*n* = 32,857) had an SVI quartile of one, 23% (*n* = 18,945) had an SVI quartile of two, 20% (*n* = 16,867) had an SVI quartile of three, and 17% (*n* = 13,766) had an SVI quartile of four.

### 3.2. Sociodemographic Differences by Year

**Figure 1:**
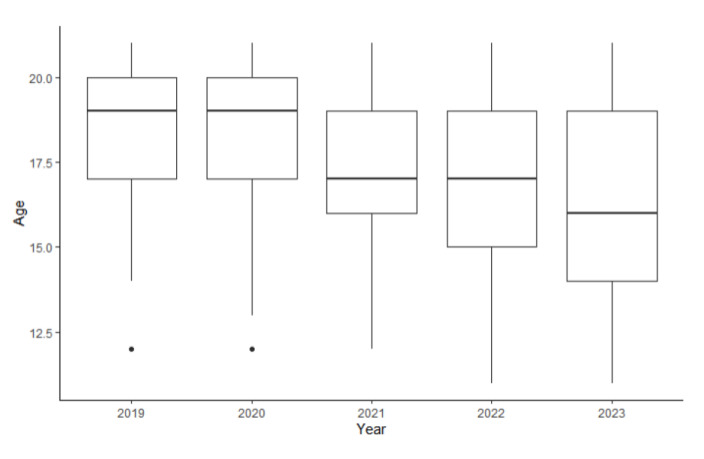
Age of patients with eating disorder diagnoses by year.

We examined the differences in the sociodemographic variables of the adolescent patients with an eating disorder diagnosis by year (Table 2). Between 2019 and 2023, the median age of the patients with an eating disorder diagnosis decreased from 19 (IQR 17–20) to 16 (IQR 14–19) years old (Figure 1). Kruskal–Wallis rank sum tests showed that the decrease in median age was significant, *p* < 0.001, with an effect size of 0.1, which demonstrated a moderate effect. Post hoc Dunn’s tests with Bonferroni correction showed that the comparisons across all groups were significant, with the largest effect size being from the comparison of 2019 vs. 2023 (r = 0.26) (Table 3).

The sex of the patients with eating disorders differed by year. A chi-square test of independence found that the relationship between sex and year was significant: *X*^2^ (12, *N* = 82,435) = 279.37, *p* < 0.001, effect size = 0.02. The adjusted standard residuals demonstrated that the differences between groups were likely related to higher levels of eating disorder diagnoses in men and boys in 2019, at 22% (*n* = 2373) and a lower proportion of diagnoses in men and boys 2023 (18%, *n* = 2355). The size of our dataset may have contributed to the statistically significant values, but the small effect size may point to less clinical significance.

**Figure 2:**
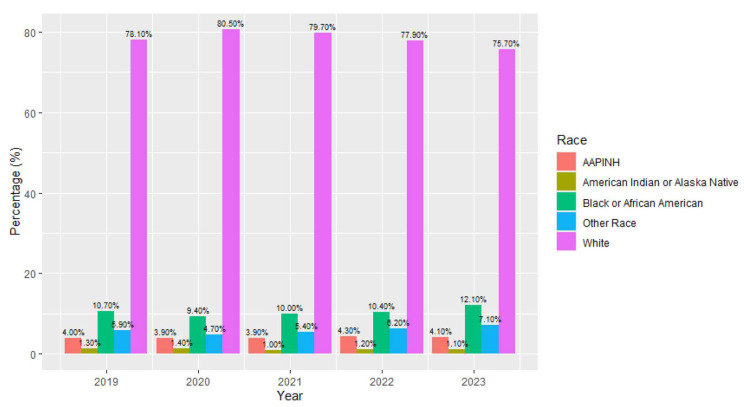
Race of patients with eating disorder diagnoses by year.

Between 2019 and 2023, there were differences in the race breakdown of the participants with eating disorder diagnoses (Figure 2). Chi-square tests of independence were performed to assess the differences between individuals of different racial backgrounds by year. The relationship between race and year was significant with a small effect: *X*^2^ (20, *N* = 82,435) = 168.41, *p* < 0.001, effect size = 0.01.The adjusted standard residuals demonstrated that the likely differences between groups were related to decreasing rates of eating disorder diagnoses in individuals who identified as White (78% (*n* = 8.549) in 2019 and 76% (*n* = 10,100) in 2023) and increasing rates of diagnoses in individuals who identified as Black or African American between 2019 (10.7% (*n* = 1176) and 2023 (12.1% (*n* = 1617).

Differences were noted in the ethnicity of the adolescent patients with eating disorders across years. The chi-square tests of independence demonstrated that the relationship between ethnicity and year was significant with a small effect: *X*^2^ (8, *N* = 82,435) = 103.97, *p* < 0.001, effect size = 0.01. The adjusted standard residuals demonstrated that the likely differences between groups were attributed to the higher proportions of individuals identifying as Hispanic/Latino in 2022 and 2023 compared to in 2020 and 2021.

**Figure 3:**
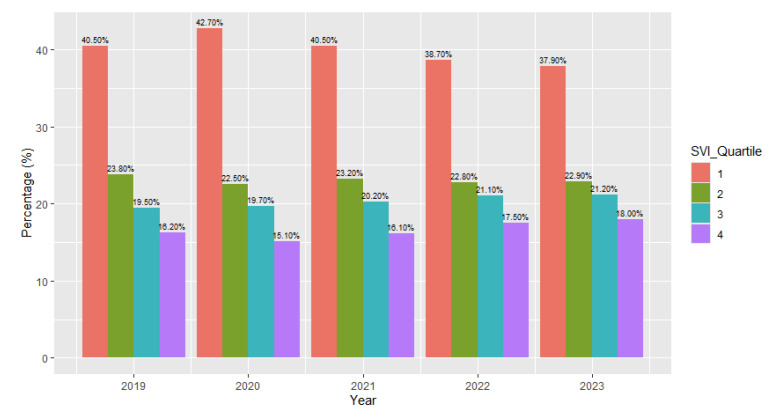
Social vulnerability index (SVI) of patients with eating disorder diagnoses by year.

There were notable differences in the SVI breakdown of the participants with eating disorder diagnoses between years in our dataset (Figure 3). The chi-square tests of independence demonstrated that the relationship between the SVI and year was significant with a small effect: *X*^2^ (12, *N* = 82,435) = 118.52, *p* < 0.001, effect size = 0.01. The adjusted standard residuals demonstrated that the likely differences between groups were attributed to higher levels of individuals with a higher SVI in 2022 (the third quartile being 21.1% (*n* = 5374) and the fourth quartile being 17.5% (*n* = 4459)) and 2023 (the third quartile being 21.2% (*n* = 2827) and the fourth quartile being 18% (*n* = 2401)).

**Figure 4:**
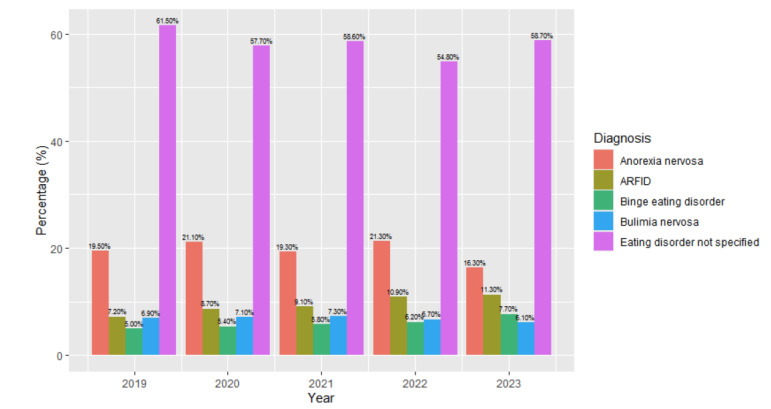
Eating disorder diagnoses by year.

Between 2019 and 2023, there were differences in the proportions of the types of eating disorder diagnoses (Figure 4). Chi-square tests of independence were performed to assess the differences between individuals with different diagnoses by year. The relationship between type of eating disorder and year was significant with a small effect: *X*^2^ (16, N = 82,435) = 468.29, *p* < 0.001, effect size = 0.02. The adjusted standard residuals demonstrated that the differences between groups was likely related to higher levels of diagnoses of eating disorder not specified in 2019 (61%, *n* = 6727), lower levels of eating disorder not specified in 2022 (55%, *n* = 13,994), higher levels of anorexia nervosa diagnoses in 2022 (21%, *n* = 5445), and higher levels of ARFID diagnoses in 2023 (11.3%, *n* = 1.507). These findings highlight that ED diagnoses became more specific between 2019 and 2023. 

### 3.3. Sociodemographic Differences by Eating Disorder Diagnosis

**Figure 5:**
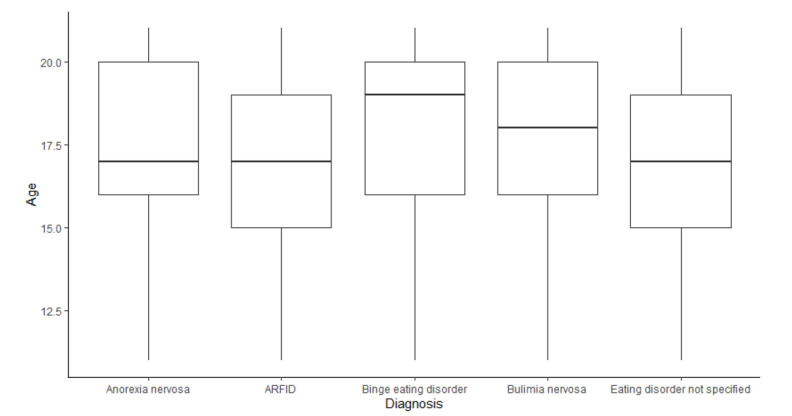
Age breakdown by diagnosis type.

We examined the differences in the sociodemographic variables of the adolescent patients with different eating disorder diagnoses (Table 4). The median age of the patients differed across diagnoses (Figure 5). The median age of patients diagnosed with binge eating disorder and bulimia nervosa (19, IQR 16–20) was higher than the median age of patients diagnosed with anorexia nervosa (17, IQR 16–20), ARFID (17, IQR 15–19), and eating disorder not specified (17, IQR 15–19). A Kruskal–Wallis rank sum test was performed and showed that the difference in median age between eating disorder diagnosis groups was significant (*p* < 0.001, effect size *r* = 0.024) with a small effect. The post hoc Dunn’s test demonstrated that the pairwise comparisons were significant across all groups except between binge eating disorder and bulimia nervosa (Table 5). The largest effect size was noted from the comparison of ARFID vs. binge eating disorder (*r* = 0.11)

There were differences in the sex among the patients with different eating disorder diagnoses. There were higher proportions of males with ARFID diagnoses (32%, *n* = 2570), binge eating disorder (22%, *n* = 1107), and eating disorder not specified (17%, *n* = 8240) compared to those with anorexia nervosa (9.1%, *n* = 1473) and bulimia nervosa (8.9%, *n* = 498). Chi-square tests of independence demonstrated that the relationship between diagnosis and sex was significant with a small effect: *X*^2^ (12, *N* = 82,435) = 103.97, *p* < 0.001, effect size = 0.05. The adjusted standard residuals demonstrated that the likely differences between groups were attributed to higher proportions of individuals identifying as male with diagnoses of ARFID, binge eating disorder, and eating disorder not specified compared to those with diagnoses of anorexia and bulimia nervosa.

**Figure 6:**
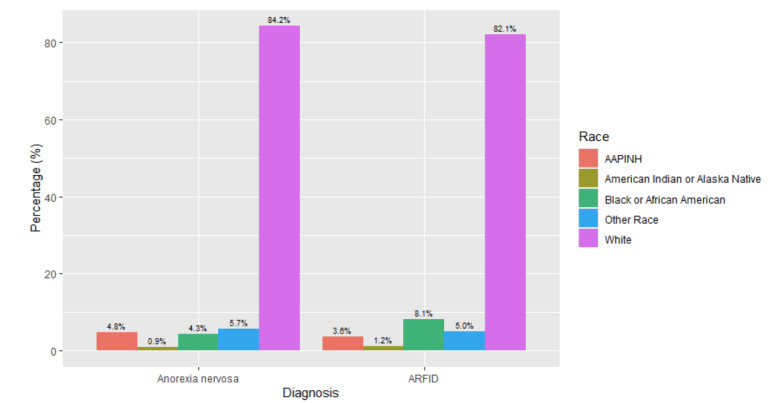
Race breakdown by diagnosis type (anorexia nervosa and ARFID).

**Figure 7:**
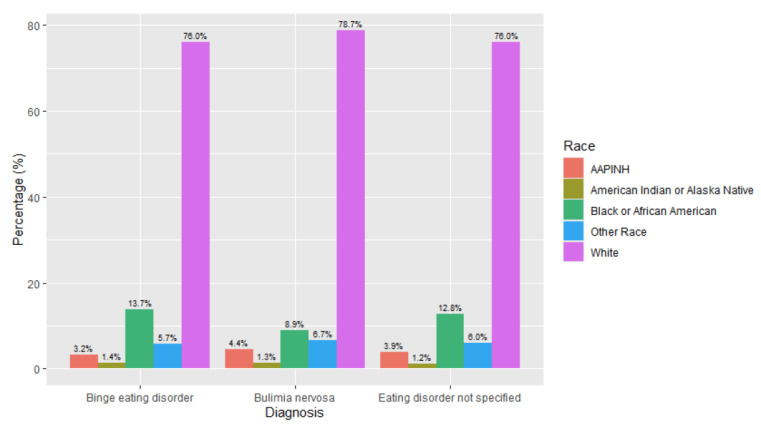
Race breakdown by diagnosis type (binge eating disorder, bulimia nervosa, and eating disorder not specified).

There were differences noted in race between patients with different eating disorder diagnoses (Figure 6 and Figure 7). There were higher levels of binge eating disorder diagnoses (14%, *n* = 685) and eating disorder not specified (13%, *n* = 6.093) in adolescent patients who identified as Black or African American. Chi-square tests of independence demonstrated that the relationship between diagnosis and race was significant with a small effect: *X*^2^ (16, *N* = 82,435) = 1150.7, *p* < 0.001, effect size = 0.03. The adjusted standard residuals demonstrated that the likely differences between groups were attributed to higher proportions of individuals identifying as African American or Black having diagnoses of binge eating disorder and eating disorder not specified, while individuals who identified as White were more likely to have diagnoses of anorexia and bulimia nervosa.

There were differences noted in the ethnicity of patients with different eating disorder diagnoses. The proportion of patients who identified as Hispanic was higher in those who had bulimia nervosa (17%, *n* = 948) and eating disorder not specified (16%, *n* = 7413), compared to those with anorexia nervosa (14%, *n* = 2199), ARFID (13%, *n* = 1044), and binge eating disorder (15%, *n* = 739). The chi-square tests of independence demonstrated that the relationship between diagnosis and ethnicity was significant with a small effect: *X*^2^ (8, *N* = 82,435) = 103.97, *p* < 0.001, effect size = 0.01. The adjusted standard residuals demonstrated that the likely differences between groups were attributed to higher proportions of individuals identifying as Hispanic having diagnoses of bulimia and eating disorder not specified.

**Figure 8:**
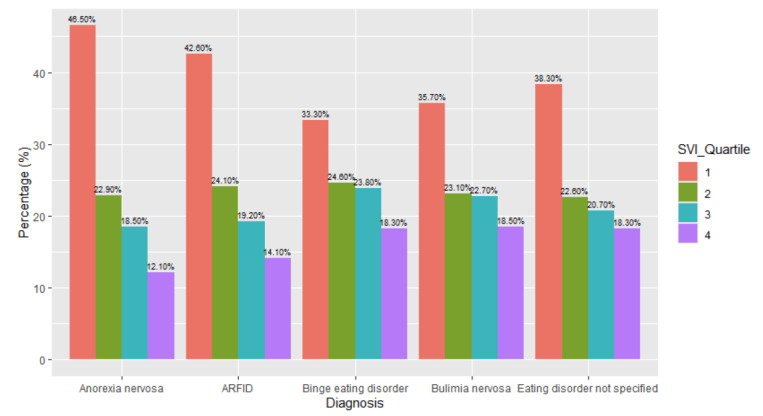
Social vulnerability index (SVI) breakdown by diagnosis type.

The differences in patients’ SVIs with different eating disorder diagnoses were noted (Figure 8). There were higher proportions of individuals who had an SVI quartile of one (46%, *n* = 7549), while patients with an SVI quartile of three were more likely to have a diagnosis of binge eating disorder (24%, *n* = 1187) and bulimia nervosa (23%, *n* = 1275). The chi-square tests of independence demonstrated that the relationship between the diagnosis and SVI was significant with a small effect: *X*^2^ (12, *N* = 82,435) = 723.16, *p* < 0.001, effect size = 0.03. The adjusted standard residuals demonstrated that the likely differences between groups was attributed to higher proportions of individuals with anorexia and ARFID diagnoses who were less socially vulnerable (i.e., SVI quartiles one and two) compared to individuals with binge eating disorder, bulimia nervosa, and eating disorder not specified, who were more likely to have higher social vulnerability (i.e., SVI quartiles three and four).

## 4. Discussion

This study described the sociodemographic differences amongst adolescent patients with eating disorders between 2019 and 2023. The results of this study supported our hypothesis about the prevalence of eating disorders since 2019. Our study demonstrated that the overall prevalence of eating disorder diagnoses in adolescent patients increased since the start of the COVID-19 pandemic. Other studies have reported similar trends [3,4]. We noted in our results that the median age of patients with eating disorder diagnoses was decreasing. These findings are important and concerning in that the literature notes more severe symptoms and greater difficulties with earlier diagnosis [23].

### 4.1. Screening Disparities

The findings also supported our hypothesis that eating disorder diagnoses amongst individuals from historically minoritized racial/ethnic groups and those with lower socioeconomic means have increased since the start of the COVID-19 pandemic. Despite the literature supporting eating disorder prevalence being equally distributed across racial, ethnic, and socioeconomic groups [7,10,12,24], our study demonstrated significant differences across these variables. Notably, our study population had higher proportions of patients who identified as White, female, and non-Hispanic and were less socially vulnerable. These findings could be reflective of the known screening disparities in marginalized populations [11]. Although there were higher proportions of individuals who identified as White and non-Hispanic in the overall dataset, the percentage of individuals who identified as White decreased between 2019 and 2023, while the proportion of individuals who identified as African American/Black or Hispanic increased during that same period. Additionally, our study demonstrated a decrease in the proportion of diagnoses of eating disorder not specified in 2023 compared to 2019, pointing to an increase in provider awareness around specificity related to ED diagnoses.

### 4.2. Diagnostic Shifts

Studies have demonstrated that although eating disorders (EDs) pose a significant threat to health, they remain under-diagnosed and undertreated in higher-weight individuals, individuals from historically minoritized racial and ethnic groups, individuals from socioeconomically challenging circumstances, and individuals who identify as male [9,11]. The differences could be related to clinicians’ unconscious biases in detecting eating disorders in certain populations, discomfort with the screening and diagnosis of eating disorders, or inadequate training around screening, diagnosis, and management [11,25,26,27,28,29]. Our study results demonstrate that significant differences between actual prevalence and rates of diagnosis may exist.

### 4.3. Sociocultural Influences

Cultural factors potentially impact diagnosis rates in certain ethnic populations [30,31]. For example, eating disorders and disordered eating are often under-reported, untreated, or associated with other comorbid conditions among African Americans and Caribbean Blacks [30]. Additionally, clinicians may be less likely to screen for or diagnose eating disorders in patients with a normal or elevated body mass index (BMI) [29,32]. Our results showed that individuals who identified as African American or Black had higher rates of binge eating disorder and eating disorder not specified diagnoses, while patients in our dataset who identified as Hispanic were more likely to be diagnosed with eating disorder not specified or bulimia nervosa. As noted in prior studies [29,32], our findings could reflect potential clinician biases in diagnosis. Additionally, complex cultural factors, including those related to acculturative stress and biculturalism, specifically in Asian American individuals, may contribute to differing levels of symptom prevalence and diagnostic differences [31].

Our study strengths include its innovative use of a large, de-identified dataset generated from EHR data, which may be more representative of the clinical situations encountered during usual care as compared to controlled scenarios in a study setting. The study population was diverse across ethnicity, socioeconomic status, and race. The population was also largely representative of the population in the United States [15]. We were able to illustrate that, despite potential barriers and biases in screening and diagnosing eating disorders in adolescent patients, there were significant increases in the prevalence and proportion of diagnoses in historically marginalized groups. We also noted a relatively large proportion of patients being diagnosed with eating disorder not specified, which may be reflective of the lack of knowledge around the screening and diagnosis of eating disorders in teens and young adults.

## 5. Limitations

Although Cosmos is a large and representative dataset, many cases had missing data and were removed prior to analysis [15]. Omitting missing variable bias may have been introduced into our analytical dataset by removing missing data. It is possible that this impacts the generalizability and interpretability of our results. Because the data compiled on Cosmos are derived from EHR data, the dataset could have limitations related to data collection and clinical workflows [16,17]. The authors employed a thoughtful aggregation of ICD-10 codes to query the Cosmos dataset. Despite these best efforts, sample selection bias could have affected our results [17]. Additionally, our study design was a retrospective cohort study. This study design has its limitations, in that we are not able to draw definitive conclusions about causality [33].

EHR data could be limited in their interpretability by user-specific documentation inconsistencies. We were also limited by the selection of the variables available within Cosmos. We were unable to obtain important information such as zip code and geographical data [15]. As noted above, in accordance with Cosmos’ guidelines, we aggregated the data for individuals from the Asian and Native Hawaiian/Pacific Islander communities, which limited our ability to draw specific conclusions for persons in these groups. Subgroup disparities may be obscured by aggregating racial data [20,21]. We were not able to include body mass index (BMI) or insurance data for our population due to large degrees of missingness, which limited our ability to draw conclusions on patients’ anthropometric and socioeconomic circumstances. BMI information would have provided important context, as prior studies have demonstrated that medical providers may be less likely to screen for EDs in patients with normal or higher BMIs [30,32,34]. Insurance data would be an important variable to consider, given that the literature notes significant barriers to treatment for patients seeking eating disorder treatment with public insurance in the United States [11].

Another limitation of our analytical dataset was the lack of inclusion of sexual orientation and gender identity (SOGI) data. The documentation practices of SOGI in the EHR lack consistency, thus leading to missing data and the exclusion of this variable from the dataset [34]. The literature demonstrates that sexual and gender minority (SGM) youth have a significantly higher prevalence of eating disorders [35,36,37]. SGM youth with eating disorder diagnoses also have much higher odds of suicide attempts. The lack of inclusion of SOGI data limited our ability to draw conclusions about this vulnerable subpopulation of adolescents.

Finally, our primary analyses utilized chi-square and Kruskal–Wallis tests, which are non-parametric and do not accommodate covariates. While we recognize this as a limitation, these tests were selected due to the characteristics of the dataset. Future studies should utilize regression-based methods to adjust for covariates to obtain further insights from the data.

## 6. Conclusions

Our study demonstrates the changes in the sociodemographic factors associated with eating disorder (ED) diagnoses in adolescent patients, including an increased overall prevalence of EDs, an increase in diagnosis rates in historically marginalized populations, and a decrease in the median age at diagnosis since the start of the pandemic. When caring for adolescent patients, clinicians and organizations should consider eating disorder diagnoses across diverse patient populations. Targeted interventions should include increasing the awareness of eating disorder prevalence in adolescence, improving screening practices, and increasing training for practitioners around eating disorders. Policymakers should allocate additional resources to support individuals of lower socioeconomic means for accessing evidence-based eating disorder treatment. Policymakers should also provide support to increase the capacity of the healthcare workforce for screening, diagnosing, and treating patients with eating disorders. Healthcare policies should also include resource allocation for individuals with EDs in non-clinical settings, such as schools or public health settings, as patients may not always present to clinical care settings [38].

Further studies on the co-occurrence of mental health conditions, SOGI, substance use, family structure, geography, and media use on eating disorder prevalence should be conducted. Future efforts should be made to disaggregate racial groups and to collect SOGI information to better understand the complex needs of diverse communities. Clinicians may consider integrating ED screening into general adolescent mental health assessments.

## Figures and Tables

**Table 1:** Total population characteristics (*n* = 82,435).

Characteristics	*n* = 82,435 ^1^
Age	17 (15.0, 19.0)
Sex	
Female	68,486 (83%)
Male	13,888 (17%)
Other/unknown	61 (<0.1%)
Race	
AAPINH	3352 (4.1%)
American Indian or Alaska Native	983 (1.2%)
Black or African American	8622 (10%)
Other race	4860 (5.9%)
White	64,618 (78%)
Ethnicity	
Hispanic or Latino	12,350 (15%)
Not Hispanic or Latino	65,448 (79%)
Diagnosis	
Anorexia nervosa	16,252 (20%)
ARFID	8006 (9.7%)
Binge eating disorder	4991 (6.1%)
Bulimia nervosa	5627 (6.8%)
Eating disorder not specified	47,559 (58%)
SVI Quartile	
1	32,857 (40%)
2	18,945 (23%)
3	16,867 (20%)
4	13,766 (17%)

^1^ Median (IQR); *n* (%).

**Table 2:** Sociodemographic variables by year.

Characteristic	2019 *n* = 10,945 ^1^	2020 *n* = 12,037 ^1^	2021 *n* = 20,537 ^1^	2022 *n* = 25,510 ^1^	2023 *n* = 13,345 ^1^	*p*-Value ^2^	Effect Size (*r*) ^3^
Age	19 (1.0, 20.0)	17 (17.0, 20.0)	17 (16.0, 19.0)	17 (15.0, 19.0)	16 (14.0, 19.0)	<0.001	0.1
Sex							
Male	2373 (22%)	2104 (17%)	3046 (15%)	4010 (16%)	2355 (18%)	<0.001	0.02
Female	8572 (78%)	9933 (83%)	17,491 (85%)	21,500 (84%)	10,990 (82%)		
Race						<0.001	0.01
AAPINH	434 (4.0%)	472 (3.9%)	808 (3.9%)	1091 (4.3%)	544 (4.1%)		
American Indian or Alaska Native	140 (1.3%)	173 (1.4%)	215 (1.0%)	310 (1.2%)	143 (1.1%)		
Black or African American	1176 (11%)	1128 (9.4%)	2047 (10%)	2651 (10%)	1617 (12%)		
Other race	646 (5.9%)	572 (4.8%)	1106 (5.4%)	1591 (6.2%)	941 (7.1%)		
White	8549 (78%)	9692 (81%)	16,361 (80%)	19,867 (78%)	10,100 (76%)		
Ethnicity						<0.001	0.01
Hispanic or Latino	1530 (14%)	1587 (13%)	2925 (14%)	4156 (16%)	2145 (16%)		
Not Hispanic or Latino	8767 (80%)	9780 (81%)	16,499 (80%)	19,953 (78%)	10,401 (78%)		
Unspecified	648 (5.9%)	670 (5.6%)	1113 (5.4%)	1401 (5.5%)	799 (6.0%)		
SVI Quartile						<0.001	0.01
1	4436 (41%)	5143 (43%)	8316 (40%)	9874 (39%)	5058 (38%)		
2	2602 (24%)	2709 (23%)	4756 (23%)	5803 (23%)	3059 (23%)		
3	2135 (20%)	2368 (20%)	4154 (20%)	5374 (21%)	2827 (21%)		
4	1772 (16%)	1817 (15%)	3311 (16%)	4459 (17%)	2401 (18%)		
Diagnosis						<0.001	0.02
Anorexia nervosa	2130 (19%)	2534 (21%)	3956 (19%)	5445 (21%)	2173 (16%)		
ARFID	788 (7.2%)	1053 (8.7%)	1863 (9.1%)	2790 (11%)	1507 (11%)		
Binge eating disorder	543 (5.0%)	655 (5.4%)	1193 (5.8%)	1574 (6.2%)	1021 (7.7%)		
Bulimia nervosa	757 (6.9%)	853 (7.1%)	1487 (7.2%)	1707 (6.7%)	811 (6.1%)		
Other eating disorder	6727 (61%)	6942 (58%)	12,038 (59%)	13,994 (55%)	8833 (59%)		

^1^ Median (IQR); *n* (%). ^2^ Kruskal–Wallis rank sum test; Pearson’s chi-squared test (%); ^3^ eta^2^; Cramér’s V.

**Table 3:** Pairwise comparisons of year for age (post hoc Dunn’s tests with Bonferroni correction).

Comparison	Test Statistic (Z)	*p*-Value (Uncorrected)	*p*-Value (Adjusted)	Effect Size (*r*) ^1^
2019 vs. 2020	−20.77	<0.001	<0.001	0.07
2019 vs. 2021	−43.98	<0.001	<0.001	0.15
2019 vs. 2022	−63.97	<0.001	<0.001	0.22
2019 vs. 2023	−75.96	<0.001	<0.001	0.26
2020 vs. 2021	−21.43	<0.001	<0.001	0.07
2020 vs. 2022	−47.49	<0.001	<0.001	0.17
2022 vs. 2023	−56.1	<0.001	<0.001	0.2
2021 vs. 2022	−29.77	<0.001	<0.001	0.1
2021 vs. 2023	−41.29	<0.001	<0.001	0.14
2022 vs. 2023	−16.85	<0.001	<0.001	0.06

^1^ eta^2^.

**Table 4:** Sociodemographic variables by diagnosis.

Characteristic	Anorexia Nervosa *n* = 16,238 ^1^	ARFID *n* = 8001 ^1^	Binge Eating Disorder *n* = 4986 ^1^	Bulimia Nervosa *n* = 5615 ^1^	Other Eating Disorder *n* = 47,534 ^1^	***p*** **-Value ^2^**	**Effect Size** **(*r*) ^3^**
Age	17 (16.0, 20.0)	17 (15.0, 19.0)	19 (16.0, 20.0)	18 (16.0, 20.0)	17 (15.0, 19.0)	<0.001	0.02
Sex							
Male	1473 (9.1%)	2570 (32%)	1107 (22%)	498 (8.9%)	8240 (17%)	<0.001	0.05
Female	14,765 (91%)	5431(68%)	3879 (78%)	5117 (91%)	39,294 (83%)		
Race						<0.001	0.03
AAPINH	782 (4.8%)	289 (3.6%)	161 (3.2%)	250 (4.5%)	1867 (3.9%)		
American Indian or Alaska Native	153 (0.9%)	97 (1.2%)	68 (1.4%)	71 (1.3%)	592 (1.2%)		
Black or African American	692 (4.3%)	651 (8.1%)	685 (14%)	498 (8.9%)	6093 (13%)		
Other Race	931 (5.7%)	398 (5.0%)	282 (5.7%)	377 (6.7%)	2868 (6.0%)		
White	13,680 (84%)	6566 (82%)	3.79 (76%)	4419 (79%)	36,114 (76%)		
Ethnicity						<0.001	0.01
Hispanic or Latino	2199 (14%)	1044 (13%)	739 (15%)	948 (17%)	7413 (16%)		
Not Hispanic or Latino	13,113 (81%)	6558 (82%)	3952 (79%)	4340 (77%)	37,437 (79%)		
Unspecified	926 (5.7%)	399 (5.0%)	295 (5.9%)	327 (5.8%)	2684 (5.6%)		
SVI Quartile						<0.001	0.03
1	7549 (46%)	3408 (43%)	1662 (33%)	2004 (36%)	18,204 (38%)		
2	3715 (23%)	1931 (24%)	1224 (25%)	1296 (23%)	10,763 (23%)		
3	3001 (18%)	1537 (19%)	1187 (24%)	1275 (23%)	9858 (21%)		
4	1973 (12%)	1125 (14%)	913 (18%)	1040 (19%)	8709 (18%)		

^1^ Median (IQR); *n* (%). ^2^ Kruskal–Wallis rank sum test; Pearson’s chi-squared test (%). ^3^ eta^2^; Cramér’s V.

**Table 5:** Pairwise comparisons of diagnosis for age (post hoc Dunn’s test with Bonferroni correction).

Comparison	Test Statistic (Z)	*p*-Value (Uncorrected)	*p*-Value (Adjusted)	Effect Size (*r*) ^6^
AN ^1^ vs. ARFID ^2^	−26.82	<0.001	<0.001	0.09
AN vs. BED ^3^	11.16	<0.001	<0.001	0.04
AN vs. BN ^4^	8.76	<0.001	<0.001	0.03
AN vs. Other ED ^5^	−14.84	<0.001	<0.001	0.05
ARFID vs. BED	30.31	<0.001	<0.001	0.11
ARFID vs. BN	28.83	<0.001	<0.001	0.1
ARFID vs. Other ED	19.15	<0.001	<0.001	0.07
BED vs. BN	−2.32	<0.001	<0.001	0.01
BED vs. Other ED	−21.19	0.02	0.2	0.07
BN vs. Other ED	−19.17	<0.001	<0.001	0.07

^1^ Anorexia nervosa, ^2^ avoidant-restrictive food intake disorder, ^3^ binge eating disorder, ^4^ bulimia nervosa; ^5^ other eating disorder, ^6^ eta^2^.

## Data Availability

The original contributions presented in this study are included in this article. The datasets presented in this article are not readily available because the datasets are maintained by the Epic Systems Corporation. Requests to access the datasets should be directed to the Epic Systems Corporation.
